# More Than a Methanotroph: A Broader Substrate Spectrum for *Methylacidiphilum fumariolicum* SolV

**DOI:** 10.3389/fmicb.2020.604485

**Published:** 2020-12-14

**Authors:** Nunzia Picone, Sepehr S. Mohammadi, Annemiek C. Waajen, Theo A. van Alen, Mike S. M. Jetten, Arjan Pol, Huub J. M. Op den Camp

**Affiliations:** ^1^Department of Microbiology, Institute for Water and Wetland Research, Radboud University, Nijmegen, Netherlands; ^2^School of Physics and Astronomy, Institute for Condensed Matter and Complex Systems, Edinburgh University, Edinburgh, United Kingdom

**Keywords:** *Methylacidiphilum fumariolicum*, higher alkanes, ethane, propane, butane, thermoacidophilic, methanotroph

## Abstract

Volcanic areas emit a number of gases including methane and other short chain alkanes, that may serve as energy source for the prevailing microorganisms. The verrucomicrobial methanotroph *Methylacidiphilum fumariolicum* SolV was isolated from a volcanic mud pot, and is able to grow under thermoacidophilic conditions on different gaseous substrates. Its genome contains three operons encoding a particulate methane monooxygenase (pMMO), the enzyme that converts methane to methanol. The expression of two of these *pmo* operons is subjected to oxygen-dependent regulation, whereas the expression of the third copy (*pmoCAB3*) has, so far, never been reported. In this study we investigated the ability of strain SolV to utilize short-chain alkanes and monitored the expression of the *pmo* operons under different conditions. In batch cultures and in carbon-limited continuous cultures, strain SolV was able to oxidize and grow on C_1_–C_3_ compounds. Oxidation of ethane did occur simultaneously with methane, while propane consumption only started once methane and ethane became limited. Butane oxidation was not observed. Transcriptome data showed that *pmoCAB1* and *pmoCAB3* were induced in the absence of methane and the expression of *pmoCAB3* increased upon propane addition. Together the results of our study unprecedently show that a pMMO-containing methanotroph is able to co-metabolize other gaseous hydrocarbons, beside methane. Moreover, it expands the substrate spectrum of verrucomicrobial methanotrophs, supporting their high metabolic flexibility and adaptation to the harsh and dynamic conditions in volcanic ecosystems.

## Introduction

Methane (CH_4_) is a powerful greenhouse gas, which is released to the atmosphere from both natural and anthropogenic sources. About 70–80% of CH_4_ is generated biologically and a large part of it is removed in the stratosphere and troposphere through reactions with chlorine and ^⋅^OH radicals (Le Mer and Roger, 2001). In addition, microbial methane oxidation is an important terrestrial methane sink (Conrad, 2020). Bacteria can convert CH_4_ to methanol aerobically using the enzyme methane monooxygenase (MMO; Conrad, 2009). Under anaerobic conditions, mostly methanotrophic archaea remove methane via reverse methanogenesis (Welte et al., 2016).

After the original discovery by Soehngen (1906), it was believed for a long time that aerobic methanotrophy was restricted to the phylum Proteobacteria, specifically in the subphyla α- and γ-Proteobacteria (Op den Camp et al., 2009). During the past decade, it was discovered that two bacterial phyla contained new methanotrophic representatives: the intra-aerobic NC10 (Raghoebarsing et al., 2006; Ettwig et al., 2010; Hu et al., 2014) and the Verrucomicrobia (Dunfield et al., 2007; Pol et al., 2007; Islam et al., 2008).

The phylum Verrucomicrobia includes highly acidophilic and mesophilic *Methylacidimicrobium* species (optimum pH 1–3: temperature 30–44°C) (Sharp et al., 2014; van Teeseling et al., 2014) and thermophilic but less acidophilic strains of the genus *Methylacidiphilum* (optimum pH 2–2.7; temperature 50–55°C) (Dunfield et al., 2007; Pol et al., 2007; Islam et al., 2008; Erikstad et al., 2019). *Methylacidiphilum fumariolicum* SolV is the most studied verrucomicrobial methanotroph to date and was initially discovered in the Solfatara volcano near Naples (Italy) (Pol et al., 2007). Strain SolV grows optimally at pH 2.7 and 55°C and it fixes CO_2_ via the Calvin cycle and N_2_ gas through a nitrogenase enzyme (Khadem et al., 2010, 2011). Methane can be used as energy source, but strain SolV is also able to use hydrogen gas as substrate, even at sub-atmospheric concentrations (Mohammadi et al., 2017; Schmitz et al., 2020). Beside methane, the Solfatara volcano in Naples (and many other volcanic areas) emits a mixture of gas that also includes ethane (C_2_H_6_, 805–1218 ppbv), propane (C_3_H_8_, 68–178 ppbv), and butane (C_4_H_10_, 8–18 ppbv) (Capaccioni and Mangani, 2001). These gases are particularly important because they could serve as additional substrate for microorganisms. Further, they are present, together with methane, in natural gas, which is commonly used in households and industries.

The oxidation of C_2_–C_4_ compounds is mainly observed in a group of bacteria that includes the genera *Corynebacterium*, *Nocardia*, *Mycobacterium*, *Rhodococcus*, *Pseudomonas* and the sulfate-reducing bacteria *Desulfosarcina/Desulfococcus* and *Desulfotomaculum* (Takahashi, 1980; Ashraf et al., 1994; Hamamura et al., 1999; Kinnaman et al., 2007; Kniemeyer et al., 2007). Recently, oxidation of alkanes under anoxic conditions was reported in archaea and catalyzed by the enzyme ethyl/methyl-coenzyme M reductase (MCR; Laso-Pérez et al., 2016; Borrel et al., 2019; Chen et al., 2019; Seitz et al., 2019; Wang et al., 2019).

In the past, the aerobic oxidation of methane and short-chain alkanes was considered to be carried out by separate groups of microorganisms (Crombie and Murrell, 2014). However, early studies already obtained indications that methanotrophs might be able to oxidize ethane, propane and butane (Leadbetter and Foster, 1960; Hazeu and de Bruyn, 1980; Shennan, 2006). In 2010, Stable Isotope Probing (SIP) experiments linked the oxidation of ethane to the family Methylococcaceae and the oxidation of propane to unclassified γ-Proteobacteria (Redmond et al., 2010). *Methylocella silvestris* (α-Proteobacteria) was the first strain that showed simultaneous growth on methane and propane. This strain contained both a soluble methane monooxygenase (sMMO) and a soluble propane monooxygenase (PrMMO) (Crombie and Murrell, 2014). Genes encoding proteins of the methylmalonyl-CoA pathway of propionate oxidation were induced during growth on propane.

The enzymes involved in aerobic hydrocarbon oxidation are usually soluble di-iron monooxygenases complexes consisting of multiple associated proteins (Shennan, 2006). sMMO also has this structure and exhibits a larger substrate range than the copper-containing particulate methane monooxygenase (pMMO; Burrows et al., 1984). However, the butane monooxygenases of *Nocardiodes* CF8 and *Mycobacterium* probably contain copper (Hamamura and Arp, 2000; Coleman et al., 2012).

The mechanism of hydrocarbon oxidation starts with the conversion of the alkane into an alcohol. More specifically, ethane is oxidized to ethanol, acetaldehyde and acetate; propane can be oxidized at the terminal or subterminal carbon atom, leading to the formation of 1-propanol, propionaldehyde, and propanoic acid in case of terminal oxidation and to 2-propanol and acetone in case of sub-terminal oxidation. Butane, instead, is oxidized to 1-butanol, butyraldehyde, and butyric acid (Shennan, 2006).

The genome of strain SolV does not encode sMMO, nor propane or butane monooxygenases, but it shows the presence of three operons for the membrane-bound pMMO. *pmoCAB1* and *pmoCAB2* operons are located in close proximity in the genome and their PmoA subunits share 84% amino acid identity. The *pmoCAB3* operon, instead, is distantly located and its PmoA3 subunit only shares 41% amino acid identity to PmoA1 and PmoA2. Experimental data have demonstrated that the expression of *pmoCAB1* and *pmoCAB2* is regulated by oxygen concentrations (Khadem et al., 2012), whereas *pmoCAB3* expression was so far not detected under any growth condition tested. One hypothesis proposes that *pmoCAB3* is of ancestral origin and its function could differ from methane oxidation (Fuerst, 2014). Therefore, the aim of this study was to test the ability of strain SolV to grow on short-chain alkanes and to investigate the expression of the three *pmo* operons.

Here we report that *M. fumariolicum* SolV can grow on ethane and propane, but not on butane. When methanol is supplied to a SolV culture with no oxygen limitation, expression of *pmoCAB1* and *pmoCAB3* could be detected. Furthermore, *pmoCAB3* expression increased upon propane addition.

## Materials and Methods

### Microorganism and Medium Composition

*Methylacidiphilum fumariolicum* strain SolV used in this study was initially isolated from the volcanic region Campi Flegrei, near Naples, Italy (Pol et al., 2007). The medium was composed of 0.2 mM MgCl_2_.6H_2_O; 0.2 mM CaCl_2_.2H_2_O; 1 mM Na_2_SO_4_; 2 mM K_2_SO_4_; 4 mM (NH_4_)_2_SO_4_, 1 mM NaH_2_PO_4_.H_2_O. A trace element solution was added resulting in the following end concentrations: 1 μM NiCl_2_, CoCl_2_, MoO_4_Na_2_, ZnSO_4_ and CeCl_3_; 5 μM MnCl_2_ and FeSO_4_; 10 μM CuSO_4_ and 40–50 μM nitrilotriacetic acid (NTA). The pH of medium was adjusted to 2.7 using 1 M H_2_SO_4_. To avoid precipitation, CaCl_2_.2H_2_O and the rest of the medium were autoclaved separately and mixed after cooling.

### Chemostat Cultivation With Methanol/Ethane

To test the consumption of ethane, a continuous culture with the standard medium containing 50 mM methanol (CH_3_OH; added through a 0.2 μm sterile filter to the medium) was used. The bioreactor was operated at 55°C with stirring at 700 rpm using a stirrer bar. The chemostat (liquid volume of 300 ml) was supplied with the medium at a flow rate of 3.9 ml h^–1^ (D = 0.013 h^–1^), using a peristaltic pump. The cell-containing medium was removed automatically from the chemostat by a peristaltic pump when the liquid level reached the sensor in the reactor. A supply of 10% O_2_ (v/v) and 5% CO_2_ (v/v) in argon (total gas flow = 10.6 ml min^–1^) was directed to the reactor by mass flow controllers through a sterile filter and sparged into the medium just above the stirrer bar. The initial pH was 2.7 and it was regulated with 0.2 M NaOH connected to the vessel by a peristaltic pump. The pH at steady state was kept at about 2.2. At steady state, while cells were grown under methanol limitation, a supply of ethane was introduced to the reactor. An O_2_ sensor (Applikon, Delft, Netherlands) in the liquid was coupled to a Biocontroller (Applikon) to monitor the dO_2_ values during growth.

### Chemostat Cultivation With Natural Gas and Methanol/Propane

Chemostat cultivations with methanol and natural gas were performed in a bioreactor (500 ml MiniBio Reactor, Applikon Biotechnology, Delft, Netherlands) with a working volume of 350 ml liquid medium. The system was run at 55°C and 1500 rpm stirring speed. pH, dO_2_, and medium level were monitored by Applikon MyControl Reactor sensors. The natural gas reactor was operated with a dilution rate of 0.024 h^–1^ (8.4 ml h^–1^ fresh medium was added) and a constant gas inflow consisting of air, N_2_, and natural gas. The natural gas mix used in this experiment consisted of 78.53% methane, 3.34% ethane, 0.46% propane, and 0.09% butane. The remaining 17.58% consisted of N_2_, O_2_, CO_2_, and trace amounts of higher alkanes. In the Netherlands, 1.8 ppm of the sulfur compound tetrahydrothiophene (THT) is added for safety reasons. During the oxygen limiting condition, 0.60 ml/min of natural gas was flowing through the bioreactor, with 2.20 ml/min air and 7.19 ml/min N_2_. Under methane limiting condition, 0.60 ml/min of natural gas was supplied to the bioreactor, with 4.40 ml/min air and 4.99 ml/min N_2_. Under both conditions, the total gas flow was 9.99 ml/min. In the experiments where propane was supplied, the reactor was operated with a dilution rate of 0.012 h^–1^ (medium contained 50 mM methanol) and a constant gas inflow consisting of air and CO_2_. Propane was added at a rate of 0.1 ml/min, resulting in a total gas flow of 1.6 ml/min. Biomass was measured as optical density at 600 nm (OD_600_) using 1 ml cuvettes with the spectrophotometer Spectronic 200 (Thermo Fisher Scientific, Waltham, MA, United States).

### Dry Weight Determination

To determine biomass dry-weight concentration, 10 ml of the culture suspension (triplicate) were filtered through pre-weighed 0.45 μm filters and dried to stable weight in a vacuum oven at 70°C.

### Batch Cultivation

The batch growth experiments were performed using 120 and 250 ml serum bottles containing 10 and 20 ml medium, respectively, with a headspace containing air, CO_2_ (10%) and CH_4_ (2%), C_2_H_6_ (4%), or C_3_H_8_ (4%). All incubations were performed at 55°C at 350 rpm.

### Gas Analysis

Alkane concentrations were measured injecting 100 μl of sample with a Hamilton glass syringe in a HP 5890 gas chromatograph (Agilent, United States) equipped with a Porapak Q column (1.8 m, ID 2 mm) and a flame ionization detector.

### Respiration Experiments

Respiration rates were determined polarographically in a respiration cell with an oxygen microsensor (RC350, Strathkelvin, Motherwell, United Kingdom) using 3 ml of whole cell suspensions of strain SolV. Methane, propane, or oxygen saturated media were injected into the respiration chamber to obtain the desired dissolved gas concentrations. The O_2_ signal was monitored and recorded using SensorTrace Basic software (Unisense, Aarhus, Denmark). The temperature and stirring rate in the respiration chamber was adjusted to 55°C and 1000 rpm, respectively. Rates were expressed as nmol O_2_ min^–1^ mg DW^–1^ and when necessary corrected for endogenous respiration.

### RNA Extraction and Transcriptome Analysis

A volume of 5 ml cell suspension was harvested from a continuous culture at steady state. After centrifugation (10,000 × *g*, 5 min) the pellet was used for RNA isolation using the RiboPure^TM^-Bacteria kit (ThermoFisher, Waltham, MA, United States) according to manufacturer’s instructions. mRNA was purified with the MegaClear kit (Ambion) and MICROBexpress^TM^ kit (Thermo Fisher Scientific, Waltham, MA, United States) according to manufacturer’s protocol. The efficiency of rRNA and small RNAs removal was analyzed using the Agilent 2100 Bioanalyzer (Agilent, Santa Clara, CA, United States). The mRNA extracted from cells grown with natural gas and with ethane was then converted to cDNA with the Ion Total RNA-Seq^TM^ Kit v2 (Thermo Fisher Scientific, Waltham, MA, United States) following manufacturer’s instructions. cDNA was amplified and purified to prepare barcoded libraries. Ion Sphere^TM^ Particles (ISPs) were used to create template positive ISPs by the Ion OneTouch^TM^ 2 instrument, which were enriched in the Ion OneTouch^TM^ ES instrument. This was performed with the Ion PGM^TM^ Template OT2 200 Kit (Ion Torrent, Life technologies). Sequencing of these templates was conducted on an Ion 318^TM^ Chip v2 using the Ion PGM^TM^ sequencing 200 Kit v2. The mRNA extracted from cells grown on propane and methanol was used to construct libraries using the TruSeq Stranded mRNA Library Prep protocol (Illumina) according to the manufacturer’s instructions. Libraries were normalized, pooled and sequenced using the Illumina MiSeq sequencing machine. For sequencing, the 150 bp single-read sequencing chemistry was performed using the MiSeq Reagent Kit v3 (Illumina, San Diego, CA, United States) according the manufacturer’s protocol. The RNA-Seq Analysis tool of the CLC Genomic Work bench software (version 10.1.1, CLC-Bio, Aarhus, Denmark) was used for sequence analysis. As a template for the transcriptome analysis, the complete genome sequence of strain SolV, available at the Microscope annotation platform (Vallenet et al., 2009), was used. Expression values were expressed as RPKM (Mortazavi et al., 2008).

Statistical analyses were performed using the DESeq2 package in R Studio (Love et al., 2014). Transcriptomics data were deposited in NCBI Bioproject database with accession number PRJEB39356.

## Results

### Oxidation of Short-Chain Alkanes by *M. fumariolicum* SolV

**FIGURE 1:**
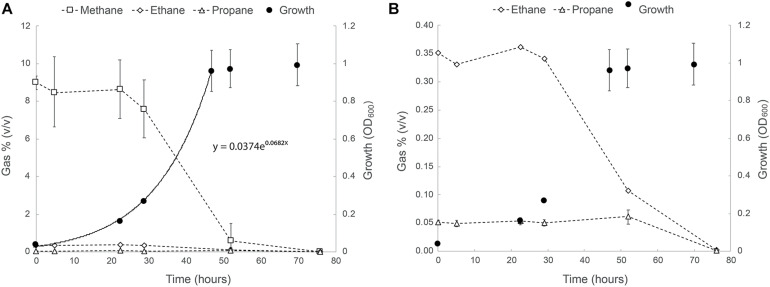
**(A)** Growth of strain SolV on natural gas (75% CH_4_, 3% C_2_H_6_, and 0.5% C_3_H_8_). **(B)** Close up of the ethane and propane consumption profiles in the same experiment. The data points are the average of three biological replicates. Error bars represent the standard deviation. Where not visible, error bars are smaller than the symbols.

The ability of strain SolV to oxidize short-chain alkanes was tested as follows: cells were incubated in bottles containing natural gas (in v/v: 75% CH_4_, 3% C_2_H_6_, and 0.5% C_3_H_8_), and this resulted in exponential growth with a doubling time of 10.2 h and a growth rate of 0.068 h^–1^ (97% of μ_*max*_; Figure 1). After 20 h, a strong drop in the methane concentrations was observed and within two days the values were below the detection limit (5 ppm). Ethane seemed to be consumed simultaneously (Figure 1). Propane consumption only started once ethane and methane became limiting (after 50 h; Figure 1). At the end of the experiment, concentrations of both ethane and propane were below detection levels. Remarkably, the sulfur compound THT seemed to be degraded as well as concluded from effluent gas analysis on a gas chromatograph equipped with a sulfur-specific flame-photometric detector (data not shown).

**FIGURE 2:**
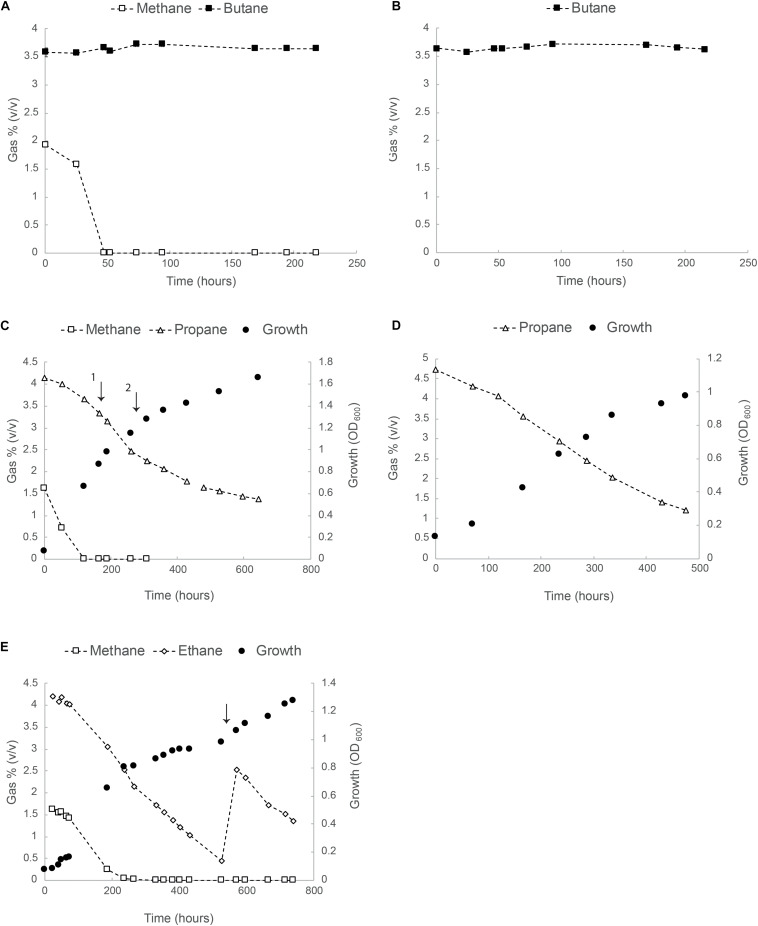
Oxidation of butane, propane and ethane by strain SolV. **(A)** Consumption of methane and butane in batch cultures. **(B)** Butane concentration over time in batch cultures containing SolV cells. In panels **(A,B)** data points represent the average over three biological replicates. All error bars indicating standard deviation (SD) are smaller than the symbols. **(C)** Batch growth of strain SolV in serum bottles with methane/propane. The first arrow indicates an addition of 4 mM NH_4_^+^ and 10 ml O_2_. The second arrow indicates an addition of extra trace element solution to avoid any limitation. **(D)** Growth of strain SolV with only propane using an inoculum from the incubation depicted in panel **(C)**. **(E)** Growth of strain SolV with methane and ethane mixture, followed by a growth only using ethane. The arrow indicates an addition of 5 ml ethane, 10 ml O_2_, 4 mM NH_4_^+^ and extra trace elements. In panels **(C–E)**, data points represent the average over two biological replicates.

In a follow up experiment, growth of strain SolV was tested in serum bottles with separate methane/alkane mixtures. Results for methane/butane (2 and 4%, v/v), methane/propane (2 and 4%, v/v) and methane/ethane (2 and 4% v/v) are shown in Figures 2A,C,E. Consumption of butane in the presence and/or absence of methane was not observed (Figures 2A,B). Propane was consumed from the start but the rate increased after methane became limiting and, although the exponential growth phase was very short, we could calculate a growth rate of 0.007 h^–1^ (about 10% of μ_*max*_ on CH_4_), equivalent to a doubling time of 102 h (Figures 2C,D).

The same batch experiments were also performed with methane/ethane (2 and 4%, v/v) at starting OD_600_ 0.07 (Figure 2E). Both gases were consumed simultaneously. Once methane was completely consumed (250 h), OD_600_ reached 0.8. At this time, about 65% of ethane was still present and growth continued till OD_600_ 1.0 and ethane became limited (Figure 2E). At this point, 5 ml ethane, 10 ml O_2_, 4 mM NH_4_^+^ and extra trace elements (enough for OD_600_ 5) were added. Growth continued to OD_600_ 1.3.

To further investigate the consumption of short-chain alkanes by SolV, cells were cultivated in a continuous system where oxygen and nutrient concentrations can be easily monitored and controlled. The chemostat was first kept under oxygen-limited conditions, using natural gas as energy source. The culture reached a steady state at OD_600_ 0.96 ± 0.09 and primarily consumed methane and ethane (Table 1). After 25 days, the air inflow was doubled, so that the amount of natural gas became limiting. The OD_600_ increased from 0.96 ± 0.09 to 1.7 ± 0.1 and the dissolved oxygen concentration from 0 to 4%. In this situation, methane consumption doubled and ethane consumption was 4.4 times higher. In addition, significant propane consumption could be detected. Butane consumption was not observed (Table 1). Ethane oxidation happened simultaneously with methane, but propane oxidation only started once methane became limiting, confirming the results of the batch experiments.

**TABLE 1:** Consumption of methane, ethane, propane, and butane by *M. fumariolicum* SolV during O_2_ and natural gas limitation.

	**O_2_ limitation^*a*^**	**Natural gas limitation^*b*^**
Methane	35.3 ± 1.4%	73.2% (73.0–73.3)
Ethane	15.5 ± 1.3%	67.9% (67.6–68.1)
Propane	0 ± 0%	10.2% (10.2–10.2)
Butane	ND^*c*^	ND^*c*^

*^*a*^Values are the average ± standard deviation (*n* = 8). ^*b*^Values are the average of two measurements with separate values between brackets. ^*c*^Butane oxidation under oxygen and methane limitation could not be determined accurately because butane concentration in natural gas were close to the detection limit of the gas chromatograph.*

The capability of multiple substrates consumption by co-metabolism provided further opportunities to establish a correlation between physiological activity and gene expression in strain SolV. The genome of strain SolV shows the presence of three *pmo* operons. To investigate their expression, a transcriptome analysis was performed under both the oxygen and natural gas limited conditions described above. Under oxygen limited conditions, *pmoCAB2* had the highest expression levels, whereas *pmoCAB1* had the highest expression under natural gas limitation (Supplementary Figure 1). These data confirmed the oxygen-dependent regulation of these operons observed before in strain SolV (Khadem et al., 2012). The third operon, *pmoCAB3*, did not show high levels of expression in both conditions.

Natural gas only contains minor amounts of ethane and propane. To investigate the consumptions of these alkanes in more detail, higher concentrations were used in a chemostat with methanol as the additional substrate. Methanol, which is as effective as methane for growth, was used instead of methane to eliminate the need for an additional gas supply.

### Oxidation of Ethane and Propane in Strain SolV

A continuous culture using methanol as an electron donor (medium flow rate = 3.9 ml h^–1^; D = 0.013 h^–1^) was established. At the steady state, when methanol was the limiting growth factor, the optical density was stable (OD_600_ = 1.03 ± 0.02) and the consumption of methanol occurred at a rate of 25.9 nmol min^–1^ mg DW^–1^. After reaching a steady state, ethane was supplied to the cells. The amount of ethane provided to the bioreactor was slowly increased from 0.36 to 7.2 μmol min^–1^. Average consumption of the supplied ethane was 50% and this resulted in an increase of OD_600_ from 1.01 to 1.71 (70% increase). The highest ethane consumption rate measured was 15.6 nmol min^–1^ mg DW^–1^, which is about 60% of the methanol consumption rate (25.9 nmol min^–1^ mg DW^–1^), supporting the increase in optical density.

**FIGURE 3:**
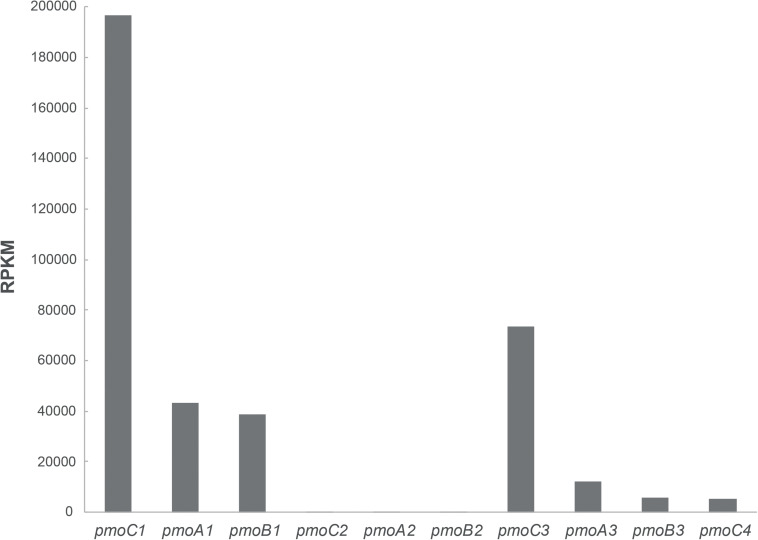
Transcriptome analysis of pMMO encoding operons in *M. fumariolicum* SolV grown on methanol and ethane. Expression values are reported as RPKM and they represent the average over two samples.

Further, cells of *M. fumariolicum* SolV grown with methanol and ethane were harvested and RNA was extracted to perform transcriptome analysis. Analysis of the expression of the *pmo* operons showed that *pmoCAB1* had the highest expression values while expression of *pmoCAB2* was hardly detectable (Figure 3). Surprisingly, *pmoCAB3* expression was noticed for the first time, suggesting that either the presence of methanol or ethane could induce its expression. The transcriptome data did not reveal any upregulation of genes that could point to the pathway used for further oxidation of ethane after conversion to acetate.

Similarly, to test the growth of strain SolV in a continuous culture grown with propane, another bioreactor using methanol as electron donor was started (D = 0.012 h^–1^). Under methanol limitation conditions, biomass reached a steady state at OD_600_ 0.84 ± 0.05. Propane was then added at a rate of 4.5 μmol min^–1^. The propane addition resulted in a new steady state at OD_600_ 1.13 ± 0.04, which equals an increase of 34% in biomass. Out of the supplied propane, only 0.1% was removed and the consumption of propane was measured at a rate of 0.46 ± 0.14 nmol min^–1^ mg DW^–1^.

Since the alkane consumption was so low, we checked the propane oxidation rate in respiration experiments using SolV cells from a continuous culture grown on methane (oxygen limited; D = 0.017 h^–1^) (Khadem et al., 2012) and we calculated a respiration rate on propane of 2.4 nmol O_2_ min^–1^ mg DW^–1^. This value corresponds to 0.48 nmol propane min^–1^ mg DW^–1^, which is in agreement with what calculated in the reactor (0.46 nmol propane min^–1^ mg DW^–1^).

**FIGURE 4:**
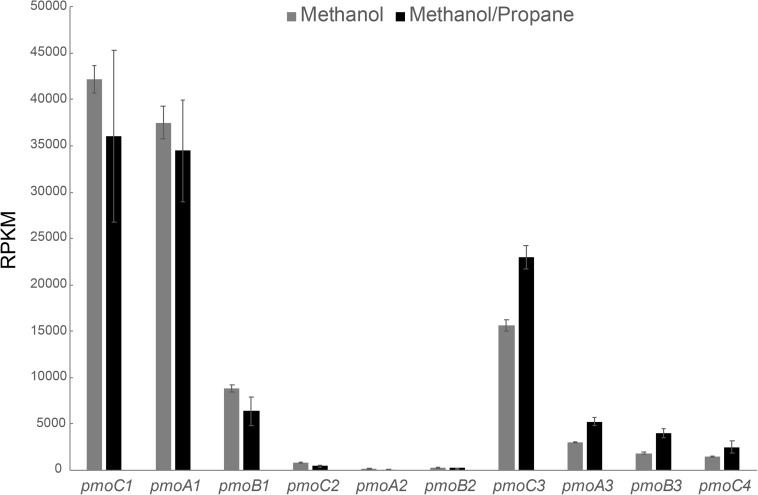
Transcriptome analysis of pMMO encoding operons in *M. fumariolicum* SolV grown on methanol (gray bars) and methanol plus propane (black bars). Expression values are reported as RPKM. Data is shown as the average over three samples and error bars represent the standard deviation.

Also for the methanol/propane culture, a transcriptome analysis was performed, but this time we analyzed both cells growing on methanol/propane and on methanol only. As shown in Figure 4, *pmoCAB1* had the highest expression in both conditions, followed by *pmoCAB3*. The *pmoCAB2* operon was not expressed. An upregulation of *pmoCAB3* and downregulation of *pmoCAB1* in presence of propane seemed to happen. However, the difference in the expression of pmoCAB1 was not statistically significant (*p* > 0.05), whereas expression of *pmoCAB3* was significantly higher (*p* = 0.00). These data shows that *pmoCAB3* expression is linked to the absence of methane or presence of methanol, but its expression levels increase when propane is supplied. The transcriptome data did not reveal upregulation of genes that could point to the pathway used for further oxidation of propane after conversion to propanoate.

## Discussion

In this study, we show that *M. fumariolicum* SolV is able to co-metabolize ethane and propane with methane or methanol, but not butane. To our knowledge, the ability to use other gaseous hydrocarbons for growth is unprecedented in a pMMO-encoding methanotroph (with no sMMO present). Most methanotrophs are highly selective, to the point that they only grow on methane and its one-carbon derivatives such as methanol, and cannot grow on complex, multi-carbon substrates like sugars or organic acids. However, the facultative methanotroph *Methylocella silvestris* that only contains sMMO (and PrMMO) is able to grow on acetate, ethanol, pyruvate, succinate, malate and propane in addition to methane and methanol (Dedysh et al., 2005; Chen et al., 2010; Crombie and Murrell, 2014). In 2010, Redmond and colleagues (Redmond et al., 2010) suggested an alternative function for *pmoA* genes, based on the ability of some *pmoA*-containing microorganisms to incorporate carbon derived from the oxidation of ethane and propane. Our study demonstrates that pMMO is indeed involved in the consumption of alkanes in strain SolV. Further, we could show that without oxygen limitation and in the presence of ethane/propane and methanol, *pmoCAB1* and *pmoCAB3* were expressed. This is the first time that *pmoCAB3* expression in SolV is ever detected. Previous work only documented the alternate expression of *pmoCAB1* and *pmoCAB2* in relation to oxygen (Khadem et al., 2012). The increased expression of *pmoCAB3* upon propane addition suggests that these genes could be involved in alkane oxidation. At the current state we cannot conclude which pMMO enzyme complex performs ethane and propane oxidation nor we can exclude that it is a combination of multiple enzymes. Further experiments, including more detailed and time-resolved RNAseq studies and expression of this operon in an alternative host, need to be performed to elucidate the exact role of *pmoCAB3* in alkane consumption.

It is expected that ethane and propane are first converted by pMMO to ethanol and propanol, respectively. The XoxF-type PQQ-dependent methanol dehydrogenase of strain SolV was shown to convert these alcohol to their aldehyde (acetaldehyde, propanaldehyde) and acid (acetate, propanoate) forms (Pol et al., 2014). The transcriptome data did not reveal upregulation of genes that could point to the further oxidation of these metabolites. Acetate could be shuttled into the central metabolism by the acetyl-CoA synthetase present in the genome of strain SolV (Mfumv2_2288). The enzymology of propane oxidation after the first two steps is poorly understood. Crombie and Murrell (2014) found an induction of the methylmalonyl-CoA pathway enzymes during growth on propane. However, the genome of strain SolV does not encode for these enzymes. However, acetyl-CoA synthetase (EC 6.2.1.1) is also know to convert propanoate into propanoyl-CoA. Metabolomic studies could shed light on the pathways involved.

The oxidation of ethane and propane in *M. fumariolicum* SolV proceeded at different rates. In particular, ethane seemed to be preferred over propane since (i) it could be oxidized simultaneously with methane, (ii) the oxidation rate was faster, and (iii) it led to a higher increase in biomass (70% vs 34%). This probably depends on the ability of pMMO of binding and converting molecules with different number of carbon atoms. Moreover, differences in the ethane oxidation rate could be noticed in different conditions. In particular, a 10-fold increase in the ethane consumption rate was calculated in the continuous culture compared to the batch incubations. This discrepancy could be due to CO_2_ or NH_4_^+^ limitations in the batch experiments. The solubility of the different alkanes at 55°C do not differ much. The values calculated from mole fractions taken from Wilhelm et al. (1977) were 0.92 mM for methane, 1.04 mM for ethane, 0.79 mM for propane, and 0.56 mM for butane.

Contrary to ethane and propane, butane consumption was not observed in strain SolV. Butane oxidation, together with propane and ethane, was detected in *Methylosinus trichosporium* OB3b. This bacterium encodes both sMMO and pMMO (Burrows et al., 1984), but its pMMO is different than the ones encoded by strain SolV. PmoA from *M. trichosporium* OB3b only shares 53% amino acid identity to strain SolV’s PmoA1, 51% to PmoA2 and 40% to PmoA3.

The sMMO-containing methanotroph *Methylococcus capsulatus* Bath also showed the ability of oxidizing C_1_–C_8_ compounds (Colby et al., 1977). The oxidation of these alkanes in *M. capsulatus* and *M. trichosporium*, however, was not linked to growth. Additionally, butane oxidation has been documented in the genera *Nocardioides, Mycobacterium, Giesbergeria, Ramlibacter, Arthrobacter, Brevibacterium* (McLee et al., 1972; Hamamura et al., 1999; Hamamura and Arp, 2000; Deng et al., 2018) and in the β-proteobacterium *Thauera butanovora* (Arp, 1999). The butane monooxygenase of *T. butanovora* (sBMO) presents high identity (38–65%) to sMMO as it contains three subunits α, β and γ encoded by *bmoX*, *bmoY* and *bmoZ* genes and a non-haem carboxylate-bridged diiron site (Sluis et al., 2002). The sBMO has a much lower affinity for methane (1.1 mM) compared to sMMO (3–13 μM) (Cooley et al., 2009). The butane degrading strain CF8, instead, seems to possess a pBMO similar to pMMO with subunit identities of 34–47% (Hamamura et al., 1999; Kinnaman et al., 2007; Sayavedra-Soto et al., 2011). A copper containing monooxygenase in *Mycobacterium* able to oxidize C_2_–C_4_ alkanes was also described (Coleman et al., 2012).

In conclusion, this study demonstrates that, beside methane and hydrogen, verrucomicrobial methanotrophs are also able to co-metabolize higher alkanes. This result is particularly important in view of the ecological role of these bacteria in the environment. Methanotrophic Verrucomicrobia appear to be not only extremely resistant to thermoacidic geothermal volcanoes, but also remarkably flexible in terms of substrate utilization. Their metabolic flexibility regarding carbon compounds could be partly provided by the differential expression of the *pmoCAB* copies in relation to the substrate available.

## Data Availability Statement

The datasets presented in this study can be found in online repositories. The names of the repository/repositories and accession number(s) can be found below: NCBI BioProject, accession no: PRJEB39356.

## Author Contributions

NP, SM, AP, MJ, and HO designed the projects and experiments. NP and AW performed the natural gas experiments. NP, SM, and AP performed the ethane/propane experiments. NP and TA sequenced and analyzed the transcriptomes. NP, AP, and HO carried out the data analysis. NP, SM, and HO wrote the manuscript. All authors contributed to revision of the manuscript, and read and approved the submitted version.

## Conflict of Interest

The authors declare that the research was conducted in the absence of any commercial or financial relationships that could be construed as a potential conflict of interest.
