# Supplementary material for: More Than a Methanotroph: A Broader Substrate Spectrum for Methylacidiphilum fumariolicum SolV
Source: Front Microbiol. 2020 Dec 14;11:604485. doi: 10.3389/fmicb.2020.604485 (PMC7768010; doi:10.3389/fmicb.2020.604485)
Supplement: Supplementary file 1 [file Image_1.pdf]

Supplementary material

More than a methanotroph: a broader substrate spectrum for *Methylacidiphilum fumariolicum* SolV

Nunzia Picone[1], Sepehr S. Mohammadi[1], Annemiek C. Waajen[2], Theo A. van Alen[1], Mike S. M. Jetten[1], Arjan Pol[1] and Huub J. M. Op den Camp[1*]

[1] Department of Microbiology, IWWR, Radboud University, Heyendaalseweg 135, NL-6525 AJ, Nijmegen, The Netherlands

[2] School of Physics and Astronomy, Institute for Condensed Matter and Complex Systems, Edinburgh University, Mayfield Rd, Edinburgh EH9 3JZ, United Kingdom

*** Correspondence:** Dr. H.J.M. Op den Camp h.opdencamp@science.ru.nl

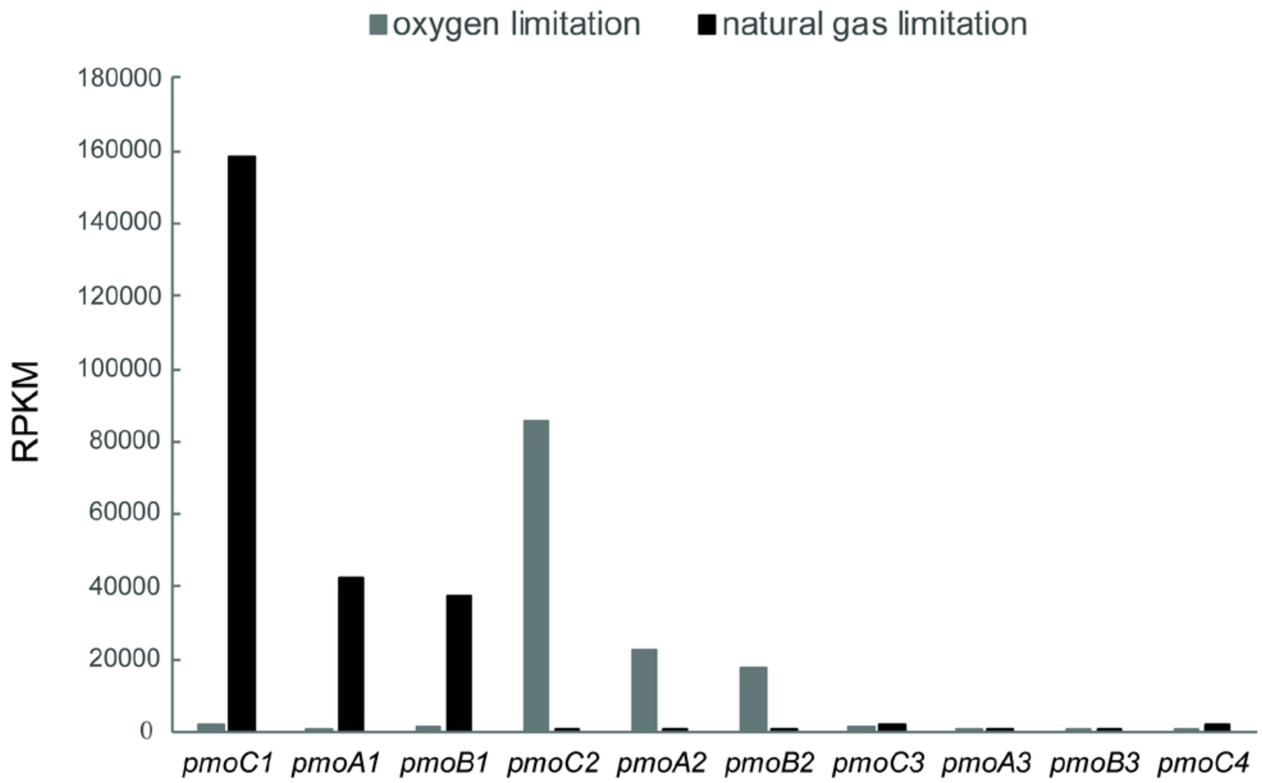

Supplementary Figure S1. Transcriptome analysis of pMMO encoding operons in *M. fumariolicum* SolV grown on natural gas. Grey bars depict the sample under oxygen limitation, the black bar depict samples under natural gas limitation. The expression values represent the average over two samples and are reported as RPKM.
